# Public Views on Models for Accessing Genomic and Health Data for Research: Mixed Methods Study

**DOI:** 10.2196/14384

**Published:** 2019-08-21

**Authors:** Kerina H Jones, Helen Daniels, Emma Squires, David V Ford

**Affiliations:** 1 Population Data Science Swansea University Medical School Swansea University Swansea United Kingdom

**Keywords:** human genome, genetic databases, public opinion, data storage, data linkage

## Abstract

**Background:**

The literature abounds with increasing numbers of research studies using genomic data in combination with health data (eg, health records and phenotypic and lifestyle data), with great potential for large-scale research and precision medicine. However, concerns have been raised about social acceptability and risks posed for individuals and their kin. Although there has been public engagement on various aspects of this topic, there is a lack of information about public views on data access models.

**Objective:**

This study aimed to address the lack of information on the social acceptability of access models for reusing genomic data collected for research in conjunction with health data. Models considered were open web-based access, released externally to researchers, and access within a data safe haven.

**Methods:**

Views were ascertained using a series of 8 public workshops (N=116). The workshops included an explanation of benefits and risks in using genomic data with health data, a facilitated discussion, and an exit questionnaire. The resulting quantitative data were analyzed using descriptive and inferential statistics, and the qualitative data were analyzed for emerging themes.

**Results:**

Respondents placed a high value on the reuse of genomic data but raised concerns including data misuse, information governance, and discrimination. They showed a preference for giving consent and use of data within a safe haven over external release or open access. Perceived risks with open access included data being used by unscrupulous parties, with external release included data security, and with safe havens included the need for robust safeguards.

**Conclusions:**

This is the first known study exploring public views of access models for reusing anonymized genomic and health data in research. It indicated that people are generally amenable but prefer data safe havens because of perceived sensitivities. We recommend that public views be incorporated into guidance on models for the reuse of genomic and health data.

## Introduction

### Background

We are witnessing a rapid expansion in the availability and use of genomic data to inform research and clinical care. The trajectory is for this trend to increase with the falling cost of whole-genome sequencing, rapid technical advances, and rise of data management facilities [1]. Work with genomic data spans the whole spectrum, from large-scale research using many thousands of records to precision medicine at the individual level. To date, the majority of genomic data have been used in large-scale research such as genome-wide association studies (GWAS) and exome-wide association studies (EWAS), among others. GWAS and EWAS are essential observational studies comparing DNA sequences and exploring variants that may be associated with a phenotypic trait. Precision medicine, where treatments and medication regimes are informed by individual genetic status, seeks to use the findings of observational studies to inform tailored clinical care. There are strong drivers for precision medicine, such as reducing the use of poorly effective interventions, thereby leading to better patient outcomes and saving costs. The UK Chief Medical Officer has expressed her dream for genomic medicine to become commonplace: “I want the NHS across the whole breadth to be offering genomic medicine - that means diagnosis of our genes - to patients where they can possibly benefit” [2]. The Department of Health program seeking to sequence 100,000 genomes has achieved this target, thereby creating a valuable data resource toward this goal [3]. The US National Institute of Health has initiated a program called All of Us, aiming to gather data from at least 1 million citizens to accelerate the introduction of precision medicine into all areas of health and health care on a large scale [4]. The Canadian government has made a major investment to advance cutting-edge developments in genomics research [5]. Successes in precision medicine are growing, but there is much further work to be done to gain the advantages and for it to become mainstream.

Between the ends of the spectrum, large scale studies using genomic data and precision medicine using individual genomic information, there is a vast range of work and many permutations of research to enable meaningful findings to be taken forward. With the exception of certain single-gene conditions such as cystic fibrosis and sickle cell anemia, the relationship between genotype and phenotype is highly complex, involving multiple genes and expression profiles. Research using genomic data in conjunction with health data (defined here as health records, phenotypic data, and lifestyle data) in condition-specific cohorts or population-level studies plays a unique role. In this type of research, it is often genomic derivatives (eg, variants and risk scores) rather than sequence data that are used with health data. Such studies allow the relationships between genomic markers, lifestyle factors, and phenotypes of interest to be explored. However, for this research to take place, genomic data collected for research studies need to be available for reuse. To be most effective, the data need to be linkable at the individual level so that genomic and wider factors can be taken into account.

There are some prevalent concerns about social acceptability and the risks posed for individuals and their kin in the use of genomic data for research. These include possible discrimination in relation to insurance coverage and employment opportunities for people with genetic conditions or high perceived risks of developing a disease [6]. However, public views on the use of genomic data have been found to be variable. The Global Alliance for Genomics and Health (GA4GH) has been running a public survey extending across numerous countries seeking views on the use of genomic data [7]. Among over 10,000 respondents, 52% felt information from DNA was different from other medical data, with 48% unsure or making no distinction. In relation to web-based data, respondents considered their banking data as needing most protection, above medical and genomic information [8]. However, although there is public engagement on various aspects of this topic, there is a lack of information about public views on models of data access. From a review on published studies using genomic data (to be published separately), we categorized 3 main data access models: open access where data are publicly available on a website, curated data released to specified researchers, and data accessed by specified researchers within a safe haven. We define a data safe haven as a secure virtual environment within which data are managed and analyzed and from which anonymized results can be released [9].

### Study Aim

To date, the majority of extant genomic data have been collected for research studies; the reuse of these datasets in deidentified form formed the focus of our study. The main aim of this study was to address the lack of information on the social acceptability of access models for reusing genomic data collected for research in conjunction with health data. This included public views on the use of the data with informed consent, without informed consent, and without consent but with notification for each of the 3 models. The reuse of any genomic data collected for clinical care and incorporated into the electronic health record (EHR) is out of scope for this study. It is assumed that the sharing of EHR data is subject to health provider mechanisms and agreements with recipient parties in line with relevant jurisdictional legislation.

## Methods

### Study Design

The study used a mixed methods approach, collecting and integrating quantitative and qualitative data. Public views were ascertained using a series of 8 workshops held between February and November 2018. Groups were selected purposively on the basis of having an interest in health care, health research, biological science, or data linkage research. The reasoning for this was 2-fold: we wanted to gain the views of groups with an interest in at least one of these areas as the study was breaking new ground; and it meant that we could tailor and reduce the explanation of concepts to meet the needs of the audience in the time available for the workshops. The groups comprised pupils at sixth-form college (in Neath Port Talbot); students at a further education college (in Pembrokeshire); university staff and students; a business professionals group; a general public consumer panel; science festival attendees, a grand round of health professionals; and University of Third Age members. All workshops were held in Wales, and where the location is not mentioned, the meetings took place on Swansea University premises. As the workshops took place at preexisting meetings with no registration process, it was not possible to control the numbers attending or influence participant selection.

Ethical approval for research with public participants was obtained from the Swansea University Medical School Research Ethics and Governance Committee. We note that in working with the public, we generally referred to genetic data rather than genomic, as genetics was a more familiar term to the participants. We did, however, explain the terms and the difference between them.

The workshops were led by KHJ and HD and ran for 1 hour. Notes of the discussions were taken (by HD and KHJ) and compared for consensus. The presentation of the study and research examples was given by KHJ, who also initiated the discussions. The information was presented in a deliberately neutral way not to preempt or influence viewpoints. The information was presented consistently across all workshops with each following the same format.

#### Initial Discussions on Public Knowledge

This involved asking the audience about the latest news item they had heard about genetics, their awareness of genetic data being used in research, and how they believe the data are used.

#### Introduction to the Study

This included describing the study purpose; the focus on the reuse in deidentified form of genetic data collected for research, as distinct from the process of data collection for clinical purposes; types of genetic data and wider health data; and how data can be accessed in terms of the 3 main models (open access, released externally, or within a data safe haven). We included a brief explanation of genetic data sequence and derivatives such as traits, variants, and risk scores.

Examples of organizations operating differing access were genetic and health data made openly available in the Personal Genome Project [10], UK Biobank collates and releases anonymized linked genetic and health data to approved researchers for specific studies [11], and the Sax Institute provides access to anonymized genetic and health data to approved researchers for specific studies within a data safe haven [12]. It was explained that, although data may be used in anonymous form, identifiable data are needed to process the primary research data for reuse and enable linkage to health data. Through these examples, we provided practical descriptions of each operating model, and how the ethical and other regulatory permissions needed for researchers to access the data can vary.

Participants were provided with examples of research studies that have used genetic data, particularly with health data, and arising from the commercial and noncommercial sectors. Studies included large-scale work to identify variants of interest, considering lifestyle factors in relation to the BRCA mutations, and medication monitoring based on genetics. We also included an introduction to direct-to-consumer genetic testing companies, such as Patients Like Me [13] and 23andMe [14], which provide sequencing services and personal genetic information to individuals, then retain and use the resulting data for research.

#### Discussions on Public Views

At this stage in the workshop, an open discussion was encouraged by asking the audience how they felt about these kinds of research taking place and what could be done to address any concerns they have.

#### Exit Questionnaire

Participants were asked to complete a questionnaire at the end of the workshop (Multimedia Appendix 1). This asked about knowledge and views on the use of genetic data and specifically asked about the relative acceptability of the 3 models of access. The questionnaire data were collected in anonymized form, and it gave the participants the opportunity to provide their views individually and privately.

Quantitative responses to the questionnaire were analyzed in IBM SPSS (version 22). Descriptive statistics were used to characterize the respondents, and for frequencies, the chi-square test was used to assess independence between categories, and the two-proportion z-test was used to compare proportions with Bonferroni correction where appropriate [15]. Free-text qualitative responses from the questionnaire were analyzed thematically by manual assessment and comparison between members of the research team (HD and KHJ) for consensus on theme identification and data convergence. A similar thematic analysis was conducted on the topics arising in the open discussions. Most analyses were based on the questionnaire responses, with the information from the open discussions being used more generally for context setting.

## Results

### Overview

The initial discussions (step 1) on public knowledge raised topics from news stories such as *3-parent* babies and designer babies, heritable genetic conditions such as Huntingdon disease, the potential for cancer research, gene editing, and invasive testing of embryos for genetic problems. Participants perceived the great value and opportunities becoming available with the increasing use of genetic data. These benefits were reiterated in the open discussion (step 3), but participants also drew attention to various concerns about privacy and confidentiality depending on data use and parties concerned. These included statements such as, “I would be worried about being discriminated against (insurance, work etc.),” “I am fearful of data in the hands of commercial companies,” and “I’m concerned about legislative and attitudinal ‘creep’—what we enforce now will, no doubt creep over time.” In terms of what could be done to address their concerns, participants highlighted the need for robust governance, data anonymization, data security, and greater transparency in data use.

### Characterizing the Respondents

Information about the attendees was collected in part A of the questionnaire shown in Multimedia Appendix 1. There were 116 respondents in total: 54 men and 62 women. The denominator in all percentage values is 116 unless otherwise stated. The age profile was as follows: 16 to 25 years: 18.9% (22/116); 26 to 35 years: 31.8% (37/116); 36 to 45 years: 15.5% (18/116); 46 to 55 years: 9.4% (11/116); 56 to 65 years: 6.8% (8/116); and older than 65 years: 17.2% (20/116). As the data were collected in age bands, mean age and standard deviation are not known. This profile was compared with the 2011 UK census figures [16] to gauge representativeness of the sample. The age bands are slightly different in the census but are close enough to provide an indicative measure. From this, we observed that our sample was overrepresented in the 26 to 35 year age band. Among the respondents, 50 (43.1%, 50/116) had children, and the remainder did not. Just more than half (n=58, 52%) held a degree with almost one-third (n=32, 33%) holding a degree in a biological subject. This higher rate than in the general population was to be expected because of the nature of the groups. By comparison, approximately 40% of the UK population are graduates [17].

Participants were asked to gauge their own background knowledge of genetics in 5 categories from none to very good. This was a matter of personal perception of one’s own ability, and as a general summary, the frequencies were no knowledge: 6.8% (8/116); a little: 34.4% (40/116); middling: 36.2% (42/116); good: 14.6% (17/116); and very good: 6.8% (8/116). This was examined further by taking into account the highest education level of the respondents in a biological subject and separately in any subject. All the qualifications are UK based, apart from degrees and professional qualifications, which are more generic categories: General Certificate of Secondary Education (GCSE) examinations are taken at age 16 years, Advanced Level (A level) examinations are taken at 18 years, Higher National Certificate/Diploma qualifications are often taken in post-16 Further Education colleges, and National Vocational Qualifications are work-based examinations. When all the categories of education level in Q5a or Q5b were included, there was no association between attainment level and self-reported knowledge. This may have been because of the variability between the categories of qualification. However, when the categories were restricted to those known to be hierarchical (GCSE, A level, and degree), attainment in a biological subject was found to be associated with higher self-reported knowledge (chi-squared *P*=.02). The relationship between self-reported knowledge and attainment across all subjects remained insignificant.

### Respondents’ Views on Data Use

The majority (78/116, 67.2%) of questionnaire respondents placed a high or very high value on using genetic data for research, with only 8 people (7/116, 6.8%) considering the value to be low or very low. No specific definition of value was presented to allow participants to make their own interpretations. Placing higher value on the use of genetic data was associated with higher attainment in a hierarchical biological subject (chi-square *P*=.005) and all subjects (chi-square *P*=.02).

There was no association between levels of value of using genetic data and levels of concern across all respondents. The most frequent level of concern about the use of genetic data for research was moderate, with 53/116 people (48%) indicating this response, 38/116 people (34%) showing low or very low concern, and 20/116 people (19%) having high or very high concern. The level of concern was not associated with educational attainment in either a biological subject or other subjects. It was also not associated with age or workshop attended. It was, however, associated with gender, with women showing higher levels of concern than men (chi-squared *P*=.01). A variety of themes emerged from the free-text responses in relation to causes for concern (Table 1), with the most frequent being misuse of data tied with concerns about information governance. Respondents noted that their concerns would be allayed if they could be assured of appropriate data use, data security, and proper information governance to avoid the data falling into the hands of agents who might discriminate against them.

**Table 1:** Concerns about the use of genetic data for research (N=116).

Type of concern	Respondents, n (%)	Percentage of total concerns
Data misuse	30 (25.9)	23
Information governance	30 (25.9)	23
Purpose of use	23 (19.8)	18
Discrimination	22 (19.0)	17
Security	16 (13.8)	13
Disclosure risk	7 (6.0)	6

Respondents’ concerns about the use of genetic data for research are given in themed areas. Each participant was asked to provide up to 3 concerns, which were then grouped into themes.

**Figure 1:**
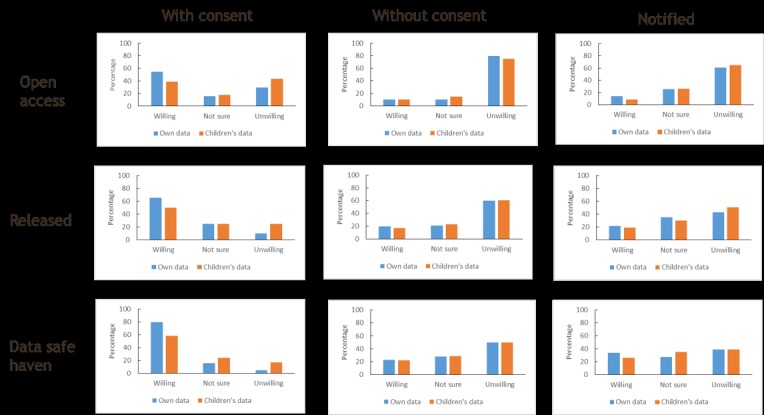
Participant views on access to their and their children’s genetic data in each of the three main access models.

The questionnaire asked participants to provide their views on access to their genetic data in each of the 3 main access models: openly accessible on a website, released to researchers, and access within a data safe haven. For each option, they were asked to indicate whether they were willing, not sure, or unwilling in relation to whether they were consented, not consented, or not consented but notified. The results of analyzing these categorical data are shown in Figure 1. Participants showed a preference for informed consent over the other options and a preference for data use within a safe haven compared with other access models. A similar, but more cautious, pattern was observed for the use of data pertaining to participants’ children. Interestingly, less than 5% of respondents stated they would be unwilling for their genetic data to be reused in a data safe haven if they were asked for consent to do so.

For each model, attendees indicated their willingness for their data to be used with consent, without being consented, and notified.

The significance of the apparent differences was tested by comparing the proportions of respondents willing for their data to be reused across consent options within each data access model (ie, intramodel seen horizontally in Figure 1). Similarly, we compared the proportions willing between models of data access (ie, intermodel seen vertically in Figure 1). We repeated these 2 analyses for willingness for children’s data reuse. We also compared proportions willing for the use of their own and their children’s data by access model for a given consent status.

### Intramodel Comparison of Data Access Preferences

We found that there was a significant difference between willingness for the reuse of respondents’ genetic data with versus without consent and with consent versus notified within all 3 access models. This was the case with and without the Bonferroni correction. There was, however, no significant difference in the proportion of respondents willing for their data to be reused between the options of without consent and notified. The same pattern was observed in their willingness for their children’s data to be reused (Table 2). This indicates that the respondents’ preferences are to be consented to the reuse of genomic data collected for research. It was not considered appropriate for reuse of genomic research data to proceed without consent, nor sufficient to be merely notified.

**Table 2:** Intramodel comparison: with consent, without consent, and notified.

Consent status—*access model*	With consent: without consent		With consent: notified		Without consent: notified	
	*P* value^a^	CI	*P* value^a^	CI	*P* value^a^	CI
Open: own	≤.001	0.33 to 0.56	≤.001	0.29 to 0.52	.40	−0.13 to 0.05
Open: children	≤.001	0.17 to 0.41	≤.001	0.18 to 0.42	.81	−0.10 to 0.08
Release: own	≤.001	0.34 to 0.58	≤.001	0.31 to 0.56	.72	−0.09 to 0.13
Release: children	≤.001	0.27 to 0.54	≤.001	0.25 to 0.52	.70	−0.10 to 0.14
DSH^b^: own	≤.001	0.46 to 0.68	≤.001	0.34 to 0.58	.09	−0.02 to 0.24
DSH: children	≤.001	0.22 to 0.51	≤.001	0.18 to 0.47	.57	−0.01 to 0.17

^a^*P* values above .05 were not considered significant.

^b^DSH: data safe haven.

This table displays comparisons of respondent preferences between consent options within each of the 3 models of data access: openly accessible, released externally to researchers, and accesses within a safe haven. Preferences in relation to their own and children’s data are shown. The significance level is 95%. The CIs are the interval lower and upper limits for the difference in proportions using the two-proportion z-test. Frequency denominators varied between questions with none less than 100 (of 116 participants) and were used accordingly. A Bonferroni correction was applied (for 3 tests in each set, thus requiring a *P* value <.017 to remain significant at the 95% level), but in this case, it did not affect the results.

### Intermodel Comparison of Data Access Preferences

We found no significant differences in respondents’ willingness for their genomic data to be reused when the open access model was compared with the release model. The results were similarly not significant between the release model and access within a data safe haven, with the Bonferroni correction. The same pattern was seen for the reuse of children’s data. There were, however, some significant differences between the open access and data safe haven models, corresponding with the greater degree of respondent concern about data being openly accessible compared with accessed within a safe haven (Table 3).

**Table 3:** Intermodel comparison: open access, release, and within data safe haven.

Access model—consent status	Open: release		Open: DSH^a^		Release: DSH	
	*P* value^b^	CI	*P* value^b^	CI	*P* value^b^	CI
With consent: own	.11	−0.24 to 0.02	<.001	0.13 to 0.37	.02^c^	0.02 to 0.26
With consent: children	.14	−0.04 to 0.26	.01^c^	0.05 to 0.34	.27	−0.07 to 0.24
Without consent: own	.07	−0.01 to 0.19	.02^c^	0.02 to 0.23	.60	−0.08 to 0.15
Without consent: children	.21	−0.04 to 0.17	.04^c^	0.01 to 0.24	.39	−0.07 to 0.18
Notified: own	.17	−0.03 to 0.18	.001	0.08 to 0.31	.06	0.00 to 0.24
Notified: children	.06	0.00 to 0.21	.004	0.06 to 0.29	.30	−0.06 to 0.20

^a^DSH: data safe haven.

^b^*P* values above .05 were not considered significant.

^c^Not significant when Bonferroni correction applied.

This table displays comparisons of respondent preferences between access models for each of the 3 consent options: with consent, without consent, and without consent but notified. Preferences in relation to their own and children’s data are shown. The conditions for the two-proportion z-test and Bonferroni correction were as for Table 2. Some results were no longer significant when adjusting for multiple testing.

### Own Versus Childrens’ Data Reuse Comparison

The comparisons showed no significant differences in the reuse of respondents’ own against children’s data for any model when the options were without consent or without consent but notified. As can be seen from Figure 1, the proportions of respondents willing for their, or their children’s, data to be reused under these options were low. There were some differences in willingness for the reuse of respondents’ own against children’s data when the option was with consent. However, the only 1 remaining significant after Bonferroni correction was reuse within a data safe haven. This was the model favored by the respondents with a greater degree of caution seen in relation to the reuse of children’s data compared with their own (Table 4).

**Table 4:** Comparison between the use of own and children’s data by access model for a given consent status.

Consent status—*access model*	With consent—own: children		Without consent—own: children		Notified—own: children	
	*P* value^a^	CI	*P* value^a^	CI	*P* value^a^	CI
Open	.027^b^	0.02 to 0.30	.94	−0.09 to 0.09	.27	−0.04 to 0.15
Release	.033^b^	0.01 to 0.29	.62	−0.09 to 0.14	.66	−0.09 to 0.15
DSH^c^	.002	0.08 to 0.34	.92	−0.12 to 0.13	.27	−0.06 to 0.21

^a^*P* values above .05 were not considered significant.

^b^Not significant when Bonferroni correction applied.

^c^DSH: data safe haven.

Respondent preferences between the use of their own and children’s data by access models and consent option are shown. The conditions for the two-proportion z-test and Bonferroni correction were as for Table 2. Most results were not significant, and this was further reduced when the Bonferroni correction was applied.

### Respondents’ Reasons for Viewpoints

Participants elaborated on reasons for their views in relation to each model. Their free-text viewpoints provided context to the numerical data. Their main concerns included data security, protection of identity, the right to make informed choices, control over data use, who might access the data under the various models, and concern about unknown future developments. Example responses for each of the access models (openly accessible, data released externally to researchers, and data accessed within a data safe haven) from a variety of participants are given below. Information about each respondent has been included for context.

In relation to data being openly accessible, viewpoints included the following:

Potential for re-identification by unscrupulous people/organisations.

female, aged 16-25 years, no children, A level in a biological subject, professional qualification in another subject, data use: high value and moderate concern

[I am] sufficiently unhappy with the idea that it would be an active deterrent from having children at all/going abroad to have them.

male, aged 26-35 years, no children, degree in a biological subject, data use: moderate value and very high concern

It’s not my place to give info of my children to strangers for any reason.

male, aged 26-35 years, has children, degree in a biological subject, data use: very high value and low concern

...want to help with research...my DNA can’t be used to re-identify me yet.

female, aged 16-25 years, no children, GCSE in a biological subject, data use: high value, low concern

In relation to data released externally, viewpoints included the following:

I don’t know enough about it [but] I’d like to help researchers find cures and things.

female, aged 16-25 years, no children, GCSE in a biological subject, data use: moderate value, moderate concern

My primary concern is the security of data if it is sent out to researchers.

male, aged >65 years, has children, degree in a biological subject, data use: very high value, low concern

If going to researchers, then it is less likely to be abused by others online etc. who are not researchers.

male, aged 46-55 years, has children, professional qualification in a biological subject, data use: moderate value and low concern

Not sure if people could be identified

female, aged 26-35 years, no children, degree in a biological subject, data use: very high value and moderate concern

In relation to data accessed within a safe haven, viewpoints included the following:

[Data used] to what end is my main concern. But overall happier in a safer environment.

male, aged 26-35 years, no children, A level in a biological subject, degree in another subject, data use: very high value and low concern

I fully expect this to be used—missing a trick otherwise.

female, aged 36-45 years, has children, degree in a biological subject, data use: high value and moderate concern

I would want to be reassured of safeguards.

female, aged 26-35 years, has children, A level in a biological subject, degree in another subject, data use: very high value and moderate concern

Happy for my data to be used as I’m and adult and I’m told. Not for my children. They need to be able to make that decision.

male, aged 26-35 years, has children, GCSE in a biological subject, degree in another subject, data use: very high value and low concern

The viewpoints reflected the workshop discussions being premised on the use of genetic data with wider health data in line with the focus of our study, but we included a question (in the questionnaire) to ask specifically about views on genetic data use linked with health record data to clarify any additional views. Many of the participants’ viewpoints expressed were the same or similar to their previous responses; however, some expressed stronger concerns, and none were less concerned. Where additional views were given, they further polarized the preference for consent and data reuse in a safe haven.

In relation to linked genetic and wider health data being openly accessible in anonymized form, participant views included the following:

Scholars need these websites to check their daily work

Fine as long as there is consent

I would need to see what the data looks like. There are too many concerns for me to agree to this

I feel it’s not safe and I don’t want the discrimination

For the release of linked anonymized genetic and wider health data, responses included the following:

I do not mind as long as it is anonymous

Acceptable as long as there is consent and data is held securely

Dependent on the research question and access limitations

Unsure—would depend on safeguards imposed

For accessing linked anonymized genetic and wider health data in a safe haven, viewpoints included the following:

Most comfortable with this

Safe, secure, governance, auditable—okay

Data being used by verified researchers for public benefit is a good thing. Having the data kept safe and controlled is a must

I am happy for this to happen provided it is safe, not sold etc

## Discussion

### Principal Findings

This study has begun to address the lack of knowledge on the social acceptability of access models for reusing genomic data collected for research in conjunction with wider health data. This is the first known study to address this topic. As noted earlier, it does not relate to the reuse of genomic data collected for clinical care, incorporated into the health record. Our findings indicate that most public participants place a high to very high value on the reuse of genomic data. This viewpoint was associated with higher educational attainment in a biological subject but was also present across all educational subjects. Their areas of concern included data misuse, how data are governed, disclosure risk, possible discrimination, and the purpose of data use and were in accord with the large-scale survey conducted by the GA4GH [7,8]. Levels of concern were not, however, associated with educational attainment but were associated with gender with women showing greater concern than men. This might be due, in part, to the fact that women show greater levels of anxiety than men in the general population [18].

A comparison of response frequencies indicated that participants preferred to give informed consent for the reuse of their own or children’s genomic data that had been collected for research, over being notified or not consented. Tests of statistical significance between without consent and without consent but notified suggest the respondents saw little difference between these 2 options and bolstered the preference for consent. Participants favored data use within a safe haven compared with the release and open access models in terms of response frequencies. However, this difference was only significant between the open access and safe haven models of data access. Free-text responses provided information on reasons for preferences. These included the desire to support research but strong views against open access, which go some way to explaining the observed results.

Although there was little statistical significance, participants expressed more caution in relation to children’s data than their own in terms of the response frequencies on access model and consent options and their elaborations on reasons for their choices. Respondents felt it was important that children are able to make their own choices in relation to the reuse of their genomic data. In practice, this depends on the age of valid consent but supports the reconsenting of young people once that age is attained. However, we also acknowledge that this might not always be practicable and propose that participants coming of age should always be taken into account at the outset so that it can be accommodated in the research plans.

We have limited this study to public views on the reuse of genomic data collected for research because this is the main source of extant genomic data and because the sharing of data contained in the health record is subject to health provider data governance, including repurposing in line with jurisdictional legislation. There are numerous long-established enterprises across the world that reuse population health data in anonymized form for research, using datasets drawn from existing health records rather than engaging in primary data collection [19]. We recognize this as distinct from data collected for research and are not making any recommendations in relation to the work of these enterprises other than to state that an insistence on additional consent for the reuse of health record data would be impractical and highly detrimental to such research [20].

The use of genomic data through web-based open access is current practice in large-scale GWAS and EWAS because of the compute capacity required in processing the data files and the need to access data across multiple sources [21]. GWAS and EWAS often use genomic data without associated wider health data because they are concerned with profiling variants of possible significance [22]. We acknowledge the indispensable value of such studies, such as for sequence alignment and variant calling. The GA4GH has proposed a system of registered access for web-based use of genomic data with health data to facilitate the reuse of data within the bounds of consent restrictions and other ethical obligations [23]. We welcome this as an improvement, as our findings with little favor for sharing genomic data through open web-based access suggest that open access would not be socially acceptable as an extensible model for research linking genomic to wider health data. Release to specified researchers or especially access within a data safe haven was preferred and also reflects the general trend in working with linked health data [9].

### Limitations

We recognize the limitations of our study. The sample size was relatively small but included a range of ages, interest areas, and backgrounds. In the interests of privacy, we did not collect the full demographic details of our participants, and having grouped age into 10-year bands, we could not drill down further on this variable. The sample size also meant that we were limited in our options for stratified analyses. The workshops took place across South Wales, and although we do not know if public opinion on the reuse of genomic data with other health data would be significantly different in other parts of the United Kingdom or wider world, we acknowledge location as a possible limitation. As noted, we also acknowledge that a greater number of our participants were educated to degree level than the general population. However, further work could be undertaken to expand the study and address this possible source of bias in the opinions expressed. We have not included a consideration of legal and ethical requirements and how they vary, as we are preparing a further publication to do these issues justice and keep the focus of this paper on public views.

### Recommendations for Future Work

The reuse of genomic data with health data is an expanding area and one that needs much further work with the public and other stakeholders. Although our participants expressed a preference for consent, there are questions around the purpose and nature of the consent. If the data are to be reused in anonymized form, then strictly speaking, consent is not required for that reuse. But identifiable data are necessary to create anonymized data, thus calling for consent for data processing into anonymized form. This would need to be made clear on the participant information sheet and in the consent process.

We recommend that consent for the reuse of research data be incorporated into the consent form at data collection to avoid subsequent difficulties with reuse. The lead author has proposed this to the UK Health Research Authority for all primary research using personal data, not limited to genomic data. It is being taken forward as advice to be given to researchers by ethics committees and institutional review boards as part of the UK integrated research application system. Example wording for the participant information sheet and consent form is given in Multimedia Appendix 2. This is being piloted and has begun to see acceptance by research ethics committees [24]. This simple idea has the potential to revolutionize data reuse by avoiding the lack of appropriate consent. However, we also acknowledge that consent is not the ultimate panacea [25], particularly with the degree of unknownness in genomic data. Further work on consent for reuse needs to ensure the public properly understand this characteristic of genomic data.

Recommendation 1: The inclusion of consent to use personal data for deidentification so data can be reused for research should be incorporated into consent forms and participant information sheets for studies collecting primary data.

Biobanks commonly use a broad consent model where participants agree to a framework for future research of certain types. In the past, this involved the use of biological samples, but in this genomic era, it also involves the use of data generated from the samples, thus raising greater privacy concerns for individuals and their kin [6]. Dynamic consent usually involves recontacting individuals to ask consent for particular uses [26]. Although there seem to be pros and cons with each, it would be difficult to enact a meaningful dynamic consent model for the reuse of data in anonymized form because the whole point of reusing data in this form is to protect (and not know) identity. But this too needs further exploration with the public and other stakeholders.

Recommendation 2: Public engagement should be conducted on a range of consent models to gain viewpoints on the acceptability of models for the reuse of genomic and health data, taking into account ethical and legal issues, and practicalities such as research utility and computing constraints.

The GA4GH proposal for registered access to web-based genomic and health data is a novel development and one requiring an assessment of public views to gauge its relative acceptability. This could follow a similar model as we have used here and could take into account factors such as types of research, parties accessing the data, requirements and constraints on data processing, compute capacity, and the pros and cons of other access models.

Recommendation 3: Public engagement should be conducted on the acceptability of registered access to web-based genomic and health data, in comparison with other access options and conditions.

As the use of genomic data is still relatively new, the level of public awareness needs to be enhanced to enable people to make informed choices. This should include a balanced explanation of the known perils and promise of genetic research and precision medicine and should be conducted transparently in a 2-way dialog, acknowledging that there are unknowns. This is also true for many health professionals, hence the rise in Genomic Medicine education. We also propose that education on this subject begins early by being properly incorporated into curricula for secondary school pupils (aged 11‑18 years) to enable current and future generations to make informed choices.

Recommendation 4: Greater efforts are needed to raise awareness, engage in public dialog, and improve education about the perils and promise of genetic data research and precision medicine.

Genomic data are not singled out from other health data in data protection legislation (at least in the European Union) [27], and it is important not to bias public opinion and stifle research by exceptionalizing the risks in reusing genomic data [28]. Nonetheless, it is imperative that robust safeguards are in place so that genetic privacy and confidentiality (including in relation to kin) are secured. We propose that there is a need for a risk-based model incorporating public views into a flexible suite of controls to protect identities and maximize data utility.

Recommendation 5: Public views should be incorporated into the development of a risk-based, flexible suite of controls for accessing genomic and health data for research.

### Conclusions

There is undoubtedly great potential in the use of genomic and health data for large-scale research and precision medicine. However, concerns about social acceptability and the risks posed for individuals and their kin need to be addressed. Although there is much public engagement on health data sharing in general and on various aspects of genomic data reuse, there has been a lack of information about public views on models for accessing combined genomic and health data. To date, most extant genomic data have been collected for research studies, and these datasets formed the focus of our study. This is the first known study to explore public views of access models for reusing anonymized genomic and health data in research. It indicated that people are generally amenable but prefer data safe havens over external release to specified researchers and over open access because of perceived sensitivities. We recommend further public engagement, and that public views be incorporated into guidance on models for the reuse of genomic and health data.

## Appendix

Multimedia Appendix 1 Copy of questionnaire for Genetic Data Integration (GeDI) workshop participants.

Multimedia Appendix 2 Example wording for inclusion in the Participant Information Sheet and Consent Form for the reuse of data collected for research.

## Abbreviations

A level: Advanced Level
EHR: electronic health record
EWAS: exome-wide association studies
GA4GH: Global Alliance for Genomics and Health
GCSE: General Certificate of Secondary Education
GWAS: genome-wide association studies
