# Supplementary material for: Public Views on Models for Accessing Genomic and Health Data for Research: Mixed Methods Study
Source: J Med Internet Res. 2019 Aug 21;21(8):e14384. doi: 10.2196/14384 (PMC6727690; doi:10.2196/14384)
Supplement: Multimedia Appendix 1 [file jmir_v21i8e14384_app1.pdf]

Appendix 1: Copy of questionnaire for Genetic Data Integration (GeDI) workshop participants

(Spacing has been reduced for publication purposes. Data were referred to as genetic, rather than genomic, in the workshops as genetics was a more familiar term to the participants.)

This questionnaire is to be answered by the GeDI workshop participants at the end of the session.

Please provide answers to the following questions. Your responses will be very useful to the study team in helping us understand the public perspective on the use of genetic data in a data safe haven.

Please refer to the information sheet or feel free to ask us questions if you need any clarification.

Thank you very much for your responses.

SECTION A: About you

1) **What is your age? Please tick:**

16 to 25 years	[ ]
26 to 35 years	[ ]
36 to 45 years	[ ]
46 to 55 years	[ ]
56 to 65 years	[ ]
65+	[ ]

2) **What is your gender? Please tick:**

Male	[ ]	
Female	[ ]	
Other	[ ]	Please specify:
Prefer not to say	[ ]	

3) **Do you have children? Please tick:**

Yes	[ ]
No	[ ]

If you answered yes to question 3, please tell us about the age of your child/ren. Please tick all that apply:

0 to 4 years	[ ]
5 to 11 years (school years 1 to 6)	[ ]
11 to 18 years (school years 7 to 13)	[ ]
Over 18 years	[ ]

4) **So we know something about your background knowledge, please estimate your level of understanding about genetic data**

None [ ]

A little [ ]

Middling [ ]

Good [ ]

Very good [ ]

5) **Please tell us a little about your qualifications (please tick):**

a. What is your highest educational qualification in biology (or another biological subject)?	b. What is your highest educational qualification in any subject?
GCSE/O level [ ]<br>A level [ ]<br>HNC/HND [ ]<br>NVQ [ ]<br>Degree or above [ ]<br>Professional qualification [ ]<br>Please specify:<br>Other [ ]<br>Please specify:	GCSE/O level [ ]<br>A level [ ]<br>HNC/HND [ ]<br>NVQ [ ]<br>Degree or above [ ]<br>Professional qualification [ ]<br>Please specify:<br>Other [ ]<br>Please specify:

SECTION B: Your views on the use of genetic data for research

6) **What level of value do you place on the use of genetic data for research?**

Very low	Low	Moderate	High	Very high
☐	☐	☐	☐	☐

Please give up to 3 reasons for your answer:

1.

2.

3.

7) What level of concern do you have about the use of genetic data for research?

Very low	Low	Moderate	High	Very high
☐	☐	☐	☐	☐

Please give up to 3 reasons for your answer:

1.
2.
3.

8) What would make you less concerned? Please provide up to 3 points:

1.
2.
3.

SECTION C: Your views on <u>your (and (if relevant) your children's)</u> genetic data being accessed for research in <u>anonymised</u> form. Please tick the relevant option:

9) How do you feel about:

a) Your genetic data being included in datasets placed on a dedicated website for research?

With consent:	Without consent:	Without consent but informed:
Willing [ ]<br>Not sure [ ]<br>Unwilling [ ]	Willing [ ]<br>Not sure [ ]<br>Unwilling [ ]	Willing [ ]<br>Not sure [ ]<br>Unwilling [ ]

b) Your children's genetic data being included in datasets placed on a dedicated website for research?

With consent:	Without consent:	Without consent but informed:
Willing [ ]<br>Not sure [ ]<br>Unwilling [ ]	Willing [ ]<br>Not sure [ ]<br>Unwilling [ ]	Willing [ ]<br>Not sure [ ]<br>Unwilling [ ]

c) To help us understand your choices, please give reasons for your views below:

1.
2.
3.

10) How do you feel about:

a) Your genetic data being included in datasets accessed within a data safe haven?

With consent:	Without consent:	Without consent but informed:
Willing [ ]<br>Not sure [ ]<br>Unwilling [ ]	Willing [ ]<br>Not sure [ ]<br>Unwilling [ ]	Willing [ ]<br>Not sure [ ]<br>Unwilling [ ]

b) Your children's genetic data being included in datasets accessed within a data safe haven?

With consent:	Without consent:	Without consent but informed:

Willing [ ]<br>Not sure [ ]<br>Unwilling [ ]	Willing [ ]<br>Not sure [ ]<br>Unwilling [ ]	Willing [ ]<br>Not sure [ ]<br>Unwilling [ ]

c) To help us understand your choices, please give reasons for your views below:

1.
2.
3.

11) How do you feel about:

a) Your genetic data being included in datasets sent to researchers?

With consent:	Without consent:	Without consent but informed:
Willing [ ]<br>Not sure [ ]<br>Unwilling [ ]	Willing [ ]<br>Not sure [ ]<br>Unwilling [ ]	Willing [ ]<br>Not sure [ ]<br>Unwilling [ ]

b) Your children's genetic data being included in datasets sent to researchers?

With consent:	Without consent:	Without consent but informed:
Willing [ ]<br>Not sure [ ]<br>Unwilling [ ]	Willing [ ]<br>Not sure [ ]<br>Unwilling [ ]	Willing [ ]<br>Not sure [ ]<br>Unwilling [ ]

c) To help us understand your choices, please give reasons for your views below:

1.
2.
3.

12) **How do you feel about:**

a. Your genetic data and health record data being linked together in anonymized form for use in research?

Please provide your views in relation to your answers to Q9-11, and comment on whether your view depends on the model of data access?

- Publically accessible website
- Data safe haven
- Released to researchers

b. Your children's genetic data and health record data being linked together in anonymized form for use in research?

Please provide your views in relation to your answers to Q9-11, and comment on whether your view depends on the model of data access?

- Publically accessible website
- Data safe haven
- Released to researchers

Thank you very much for taking part. We will not use any identifying information about you in the presentation of the findings.
