# Supplementary material for: Public Views on Models for Accessing Genomic and Health Data for Research: Mixed Methods Study
Source: J Med Internet Res. 2019 Aug 21;21(8):e14384. doi: 10.2196/14384 (PMC6727690; doi:10.2196/14384)
Supplement: Multimedia Appendix 2 [file jmir_v21i8e14384_app2.pdf]

Appendix 2. Example wording for inclusion in the Participant Information Sheet and Consent Form for the reuse of data collected for research

(This would be in addition to the standard and study-specific information provided to research participants.)

For the Participant Information Sheet:

'The data you provide to us is important and may be useful to other research studies. So that your data can be used anonymously for further research in the public interest, a health service organisation will replace your identifying details with a unique anonymous code. This will enable your data to be linked to routinely-collected data, including your health records. The data can then be used for research in anonymous form in a secure environment.'

For the Consent Form

'I understand that my personal data may be de-identified by a health service organisation so that it can be used in anonymous form for further research in the public interest.'
